# FPGA-Based Multiprocessor System for Injection Molding Control

**DOI:** 10.3390/s121014068

**Published:** 2012-10-18

**Authors:** Benigno Muñoz-Barron, Luis Morales-Velazquez, Rene J. Romero-Troncoso, Carlos Rodriguez-Donate, Miguel Trejo-Hernandez, Juan P. Benitez-Rangel, Roque A. Osornio-Rios

**Affiliations:** HSPdigital-CA Mecatronica, Facultad de Ingenieria, Universidad Autonoma de Queretaro, Campus San Juan del Rio, Rio Moctezuma 249, Col. San Cayetano, 76807 San Juan del Rio, Qro., Mexico; E-Mails: bmunoz@hspdigital.org (B.M.-B.); lmorales@hspdigital.org (L.M.-V.); troncoso@hspdigital.org (R.J.R.-T.); cdonate@hspdigital.org (C.R.-D.); mtrejo@hspdigital.org (M.T.-H.); benitez@uaq.mx (J.P.B.-R.)

**Keywords:** smart-sensor, sensor network, FPGA, plastic injection molding

## Abstract

The plastic industry is a very important manufacturing sector and injection molding is a widely used forming method in that industry. The contribution of this work is the development of a strategy to retrofit control of an injection molding machine based on an embedded system microprocessors sensor network on a field programmable gate array (FPGA) device. Six types of embedded processors are included in the system: a smart-sensor processor, a micro fuzzy logic controller, a programmable logic controller, a system manager, an IO processor and a communication processor. Temperature, pressure and position are controlled by the proposed system and experimentation results show its feasibility and robustness. As validation of the present work, a particular sample was successfully injected.

## 1. Introduction

Plastic injection molding (PIM) is a complex process that involves many variables such as pressure, position, velocity, temperature, and several discrete input/output (I/O) events. In most cases, those variables are closely related, making it difficult to perform an accurate control that impacts the final product quality. Moreover, primary sensors such as thermocouples commonly have non-linear behavior and the signals are heavily noisy, with embedded electromagnetic interference and other sources. These defects demand a preprocessing stage to filter the signals of interest that will be used for further control operations. An effective strategy to improve the sensor measurement quality is the smart-sensor approach that copes with the limitations of primary sensors [1].

The plastic injection molding process consists in heating the plastic, after which the polymer is transported into the mold where the cooling is carried out and finally the product is ejected. First, the mold is closed and emptied, the injection unit is charged with material and the plastic is melted. In the following step, the polymer is injected with the screw, which acts as piston, the plastic fluid cross the mold sprue through the mold cavities. Next, the injection pressure is kept constant aiming at preventing contractions in the piece, after that, the pressure is eliminated, the cooling of the piece is ended and the mold is opened. Finally, the piece is ejected, the mold is closed and the cycle starts again. This process is widely used for plastic transformation due to its versatility for obtaining complex geometries, high production levels, low costs, low or null finishing, variety of colors, transparency and opacity [2]. The product quality depends on the careful monitoring of conditions to control the molding injection process, but this is difficult because of the non-linear dynamics and the model uncertainty [3]. In the PIM process pressure, position, velocity, and temperature are some of the most important variables that influence the product quality [4]. Therefore, many efforts have been made to improve the injection cycle, Jianming and Yixing [5] propose a fuzzy-PID controller to improve the melting process replacing the hydraulic motor with permanent magnet synchronous motors, and the proposed controller improves the response compared with a traditional PID according to the performed simulations. Moreover, temperature control along the injection pipe is very important; Lu and Tsai [6] developed an adaptable temperature control to deal with the coupling effect of four different temperature zones in the injection pipe. The proposed method includes the calculation of a recursive least-square method to estimate the system parameters and to adapt the controller under some perturbations. This control was implemented in a digital signal processor (DSP) performing an indirect self-tuning and control tasks that are robust under load disturbances and parameter variations. In addition, Shu and Shu [7] proposed a temperature control for a PIM based on a proportional, integral, and derivative neural network (PIDNN). The developed PIDNN gives similar results as a traditional PID, but it decouples the different temperature zones with good simulation results. Fuzzy controllers have been used to save energy in a PIM applied to a ram velocity controller [8] or to control the speed of the injection screw and the pressure of the injection nozzle in a retrofitted closed-loop injection molding machine [3]. Moreover, low cost processes improvement, increased product quality and use of new technologies in traditional processes are some of the benefits of retrofitted machine tools. On the other hand, independently from the control, the sensor measurement strategies are very important due to its effects on control performance.

A way to improve the sensor measurements is by using smart-sensors which commonly perform self-adjusting [1], signal filtering [9], transducer resolution increasing [10], and fused parameters estimating functions [11,12]. Furthermore, smart sensors are utilized to measure variables such as temperature [1], voltage and current [10], motion dynamics [9], kinematics [11], and many other derived variables. Mekid [13] presented an embedded network sensors using wireless temperature sensors, pressure, humidity and a three-axial accelerometer for thermal error assessment in machine tools, but using a full control via a software-based (OpenCNC) open architecture controller. Although recent research in PIM employs fuzzy and neural network approaches, most of industrial controllers use a PID driver approach implemented on a programmable logic controller (PLC) layout [14]. In the PIM process it is necessary to control many variables that depend on the PLC system architecture and this increases its complexity [15]. These calculations make the control a computational-intensive problem that limits the control performance. Hence, a multi-CPU system was proposed by Siyun *et al.* [16] using three CPUs to separate control, monitoring, and interface tasks, showing that this architecture simplifies the integration in a PIM system. An approach using intelligent systems which is an advance in the integration of systems was developed by Mattoli *et al.* [17], who propose a universal intelligent sensor interface (UISI) based on microcontrollers yielding high system flexibility, but at the expense of a limited processing capability. The use of smart sensors in industrial applications that require online monitoring has been extensively studied, and due to the high processing capability required they have been implemented into an FPGA, for example a smart-sensor [18] a dedicated PLC [19], control schemes in combination with DSP [20], among others [21,22]. FPGAs are devices that are especially useful to develop embedded system platforms to perform industrial process control [23] and intensive-computing [24]. Therefore, it is desirable to create a system that closes the gap around the smart-sensor network, intelligent control methods like fuzzy logic, and discrete control based on PLC with enough computational capabilities to achieve the complete operations of a retrofitted PIM machine with a single chip.

This work presents a novel strategy to retrofit the control of a plastic injection molding machine based on sensor network, fuzzy control strategies and reconfigurable technology. The network includes a smart-sensor processor, a programmable logic controller, several micro fuzzy logic controllers, and a microprocessor network. The system is developed into a low cost FPGA device with high computational capabilities to handle synchronized and parallel processes. An experimental validation of the proposed method in a retrofit PIM machine is carried out and the results are discussed.

## 2. System Architecture

**Figure 1.**
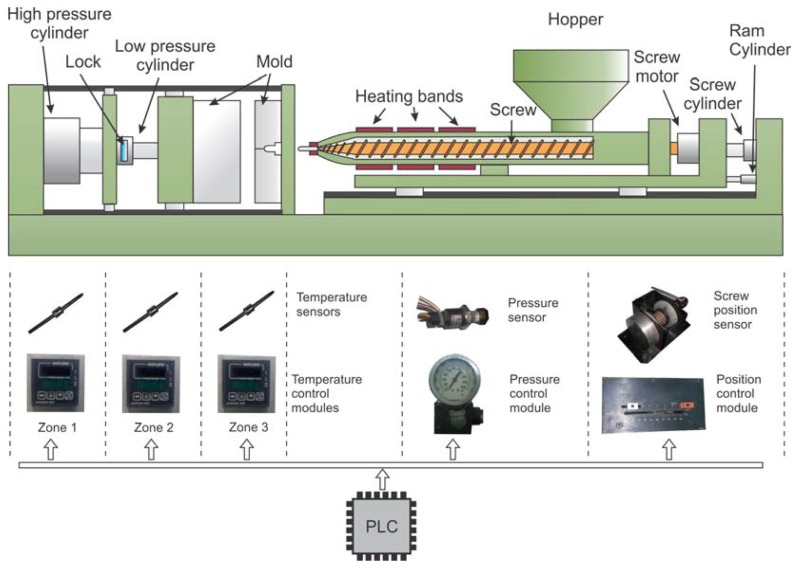
Typical control in a conventional plastic injection molding machine.

In the PIM process, it is necessary to control many variables. This fact increases the complexity of the PLC architecture and its computational load due to the variables must be controlled in parallel form. Traditionally, in a conventional plastic injection machine (Figure 1), the coordination of different analog control modules and several discrete inputs and outputs is accomplished by a PLC device. Due to the complexity of the process, the variables are controlled separately and a dedicated analog control module is utilized for each one. Different analog devices to display the temperature for each heating zone, the injection pressure and position are also utilized. On the other hand, it is common that nonlinearities in the process provoke errors when classical controllers are used due to its adaptability and robustness problems [3].

**Figure 2.**
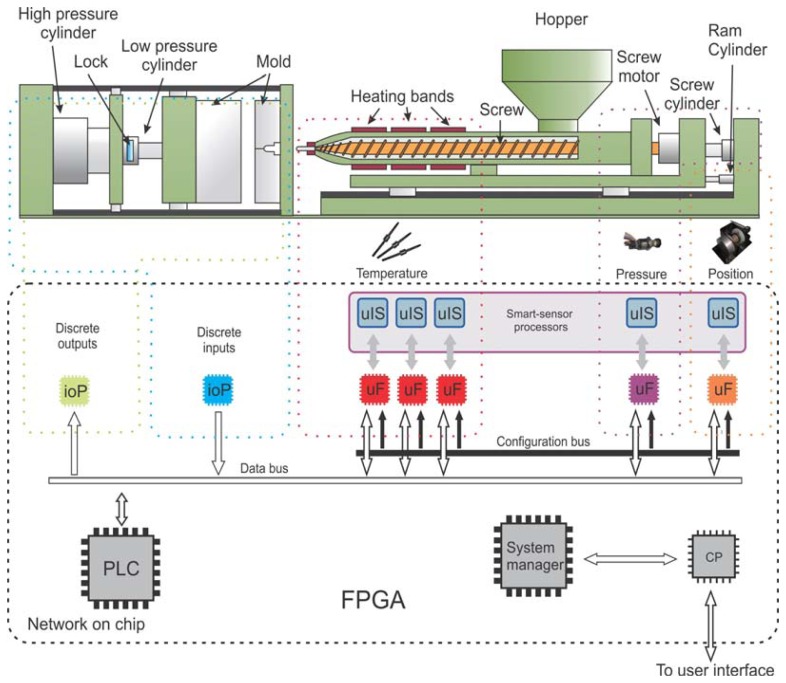
Embedded smart-sensor and controlling network architecture to control a plastic injection molding machine with six different types of embedded processors.

On the other hand, the proposed embedded smart-sensor and controlling network includes six types of embedded processors: a smart-sensor processor (uIS), a micro fuzzy logic controller (uF), a PLC, a system manager (SM), an IO processor (ioP), and a communication processor (CP). Figure 2 shows the overall architecture in the machine context.

In order to accommodate the necessary processing units in a single chip, it is essential to divide the control processes to reduce the system complexity. The PLC is the main processing unit; it controls the injection process sequence for each operation cycle. The PLC communicates with the input-output processors (ioP) and the micro fuzzy logic controller (uF) via the data bus. The PLC reads all analog inputs (*i.e.*, temperature, pressure, *etc.*) and digital inputs (*i.e.*, valves, locks, safety interrupters, *etc.*); it also updates all the digital outputs (*i.e.*, relays, coils, valves, *etc.*), and sets the reference for the fuzzy controllers in each cycle. The uFs controls the analog variables such as temperature, pressure and position. Each uF communicates with a smart-sensor processor that measures the signal of interest and controls its actuator. All processors are configured via the configuration bus, handled by the system manager (SM); it programs the PLC sequence into its internal program memory, passes the fuzzy rules to the uF, and programs the sequence in the uIS. Finally, the SM handles the communication between the PLC and the user by using a universal serial bus (USB) interface.

### 2.1. Smart-Sensor Processor

An essential unit in the proposed system is the smart-sensor processor (uIS); it is a dedicated processor that acquires and filters analog signals from the transducer, and it also includes the necessary units to operate one actuator. The smart-sensor is an interface between the real-world target process and the discrete-control processor.

**Figure 3.**
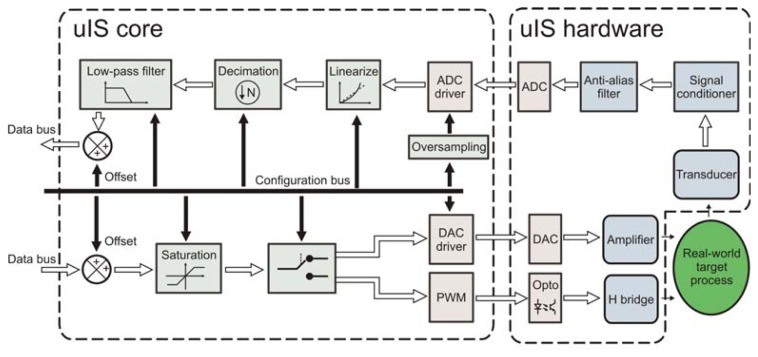
The smart-sensor processor is structured by the processing unit (uIS core) and the on board hardware elements (uIS hardware).

The uIS is composed of two parts: the hardware elements (uIS hardware) and the processing functions (uIS core), as shown in Figure 3. The uIS hardware considers the incorporated devices on the application board including: one transducer, one signal conditioner, one anti-alias filter, one analog-to-digital converter (ADC), one digital-to-analog converter (DAC), one four-quadrant pulse-width-modulator (PWM), and one opto-isolation stage for the PWM. On the other hand, the uIS core contains the digital processing tools to do the measurement properly and to direct the control signal to the actuator. The uIS core in the input signal path includes: one digital driver for a serial ADC, one oversampling unit, one decimation unit, one low-pass filter (LPF), one offset adjustment, and one linearization unit. For the output signal the uIS core includes: one offset correction unit, one saturator, one output selector, one digital driver for a serial DAC, and one PWM signal generator.

The uIS hardware receives the transducer signal, conditioned to an industrial standard of ±10 V and filtered by an anti-alias filter; then, the ADC converts the analog signal to a digital number. The DAC is directly connected to a power amplifier, while the input PWM signals are optically isolated from the H-bridge.

The ADC driver unit in the uIS core depends on the specific ADC device mounted on the board, and in the developed system an ADS7841 by Texas Instruments was used, therefore, the digital driver was implemented accordingly. If the transducer is not linear, the data must be linearized before any digital signal process could be applied. Thermocouples are well known non linear transducers; they can be linearized by polynomial approximation, depending on the thermocouple material [25]. A typical implementation of a fitting polynomial is using the nested polynomial form defined by Horner's algorithm in Equation (1), where *a*_n_ are the polynomial coefficients, *x* is the non-linear input, and y is the linearized output:

(1) $$y=a_{0}+x(a_{1}+x(a_{s}+x(a_{3}+x(a_{4}+x(\ldots a_{n-1}+a_{n}x)))))$$

Data acquisition is performed with the oversampling technique developed by Rangel-Magdaleno *et al.* [9], at the maximum ADC speed; then, the decimation unit is configured by the user depending on the process requirements. Frequently, the transducer signals need to be filtered by a LPF to improve the controller performance [26]. In addition, the LPF can be used to adjust the signal gain along with the offset adjustment to achieve the calibration of the primary sensor. The LPF is defined as a first order filter in the Equation (2), where *k*_f_ is the filter gain and *ω*_c_ is the cut-off frequency in rad/s:

(2) $$G(s)=\frac{k_{f}\omega_{c}}{s+\omega_{c}}$$

By using the bilinear transform filter, Equation (2) is converted to its discrete-time representation in Equation (3), and its difference Equation (4), where *T* represents the sampling period, *x(k)* the input signal, and *y(k)* the filter output:

(3) $$H(z)=\frac{k_{f}\omega_{c}T(z+1)}{\left(\omega_{c}T+2)z+(\omega_{c}T-2\right)}$$

(4) $$y(k)=\frac{k_{f}\omega_{c}T}{\omega_{c}T+2}x(k)+\frac{k_{f}\omega_{c}T}{\omega_{c}T+2}x(k-1)-\frac{\omega_{c}T-2}{\omega_{c}T+2}y(k-1)$$

The LPF in Equation (4) is directly implemented in hardware and it receives only three parameters: gain (*k_f_*), cut-off frequency (*ω_c_*), and sampling period (*T*). For the actuator output, the first unit adjusts offset to the data to compensate the DAC output offset. As a security feature, a saturation unit avoids the roll-over where the lower and upper saturation limits are configured by the user. Furthermore, a switch selects one of the output devices: the DAC or the PWM. The DAC driver module is designed to use a DAC7565 by Texas Instruments. The PWM used has a minimum resolution of 12 bits.

### 2.2. Micro Fuzzy Logic Controller Architecture

**Figure 4.**
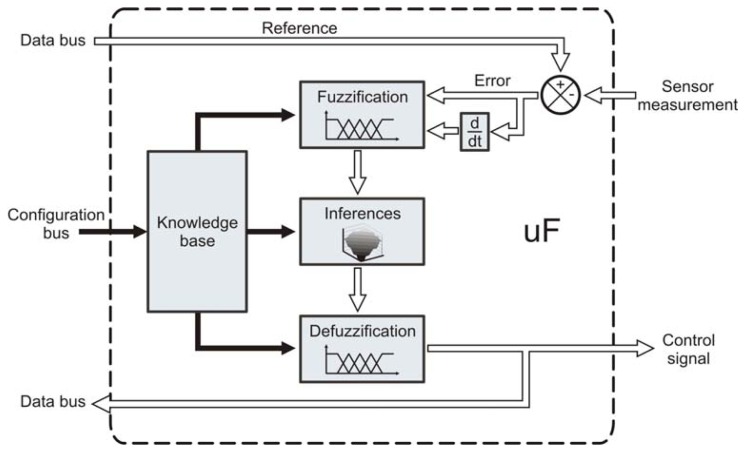
Overall micro fuzzy logic controller architecture, it shows the fuzzy logic elements and the error calculation along with its derivative, which are used in the control algorithm.

All continuous variables are controlled by utilizing a fuzzy control strategy (Figure 4), mainly due to its robustness towards non-linear effects [27]. Therefore, a special purpose processor was developed to carry out the control operations; the micro fuzzy logic controller (uF) performs a complete set of instructions for a fuzzy logic control process. The processor design yields to program the fuzzy control sets by software, which includes membership functions and knowledge base. Since the fuzzy controller is considered an expert system it requires a knowledge base provided by a human expert, this knowledge is supplied to the processor in terms of membership functions and inference rules [27].

In a fuzzy controller the input measurement coming from the smart-sensor is subtracted from the reference set-point, then, the error between the desired value and the measured value is used as input for the fuzzy logic algorithm. In this case, the proposed uF is designed as 2-input 1-output system, the inputs are the error and its derivative which is a common practice in the design of fuzzy controllers [27]. The output corresponds to the control variable applied to the actuator.

The fuzzification consists in assigning a linguistic representation to the input variable depending on the membership functions. This linguistic variable is then interpreted by an inference rule table that produces a linguistic variables output set; then, these output variables are defuzzified depending on the degree of membership to obtain a single output value. The proposed processor contemplates trapezoidal membership functions in the extremes and triangular membership functions with an overlapping of 50%. The proposed system can handle a maximum of five input membership functions for each input variable; therefore, a maximum of 25 inference rules can be implemented. For the defuzzification process the processor uses the center of gravity method, which also has a maximum of five triangular membership functions. The uF is implemented as hardware function described on VHDL code, including the hardware processor units shown in Figure 4.

### 2.3. Programmable Logic Controller Architecture

**Figure 5.**
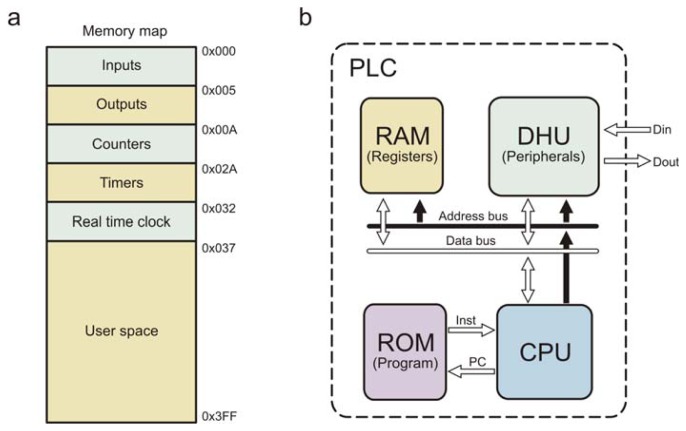
(**a**) PLC memory map. (**b**) PLC architecture.

A programmable logic controller core was developed to execute the basic commands for a PLC, it is based on an 8-bit Harvard-RISC architecture microcontroller, Figure 5(a) depicts the PLC architecture and Figure 5(b) details its memory map.

The PLC consists of one CPU and several peripherals, the CPU has a standard Harvard architecture, but the microcontrol unit (MCU) was optimized to execute the main PLC control loop automatically. The PLC has a maximum of 64 Kb of data memory and 64 Kb of program memory. This processor is designed with a direct memory access (DMA) system that allows peripherals to update its current data into the data memory. The first memory segment (0x000–0x004) is directly mapped for digital inputs, the second segment is for digital outputs (0x005–0x009), the third segment is for general purpose counters (0x00A–0x029), fourth segment is for 16-bit timers (0x02A–0x031), fifth segment is for a real-time clock (0x032–0x036), and last segment is for general purpose use (0x037–0x3FF). The instruction set for the proposed PLC was properly developed and follows a RISC philosophy.

### 2.4. System Manager Unit

The developed system is based on the DRC platform presented by Morales-Velazquez *et al.* [23], this platform defines a methodology to interconnect the hardware unit with its equivalent software client, making the communication between them transparent. By using the design tools, a register definition was implemented based on the PLC memory map and the uF memory map. The system manager (SM) is a processor that receives and decodes the messages from the user. The SM is able to hear the requests to build and send a package to the user. Each register in the system is connected to a specific function, such as, PLC memory, uF membership functions, uIS configuration, *etc.* All data and configuration information is handled by the SM.

### 2.5. IO Processor and USB Interface Units

The ioP unit is a programmable peripheral interface (PPI) unit that expands the PLC 8-bit data bus into 64 input bits and 64 output bits. Because of the large number of IO in the system, it requires some auxiliary devices controlled by the ioP; these devices are small microcontrollers which use the I2C protocol controlled by the ioP expanding the data bus. The utilized USB interface is a proprietary development of a dedicated processor that handles the USB port at full-speed. This interface has one output end-point of 64 bytes and one input endpoint of 64 bytes with a bulk transference mode.

### 2.6. Implementation Resources

This subsection details the implementations resource used by the processing units of the proposed system. The implementation device is a proprietary FPGA board, which includes eight analog inputs, eight analog outputs, 16 digital inputs, 16 digital outputs, memory, and a USB interface; the FPGA device is a Spartan3E XC3S1600E. In Table 1, it is summarized the resource used by unit in the device according to Figure 2. The proposed system uses 40% of LUTs, 8% of BRAMs, 12% of Slices, and 16% of MULTI18×18, synthetized with Xilinx ISE 12.3.

## 3. Experiment and Results

In order to validate the proposed system, in this section the experimental set up and results are presented. Due to its importance in the plastic injection molding process, experiments to regulate temperature, pressure and position by a micro fuzzy logic controller, using the uIS module configured accordingly, in a PIM machine were carried out. In the control temperature section three different heating zones are analyzed. In the same way, injection pressure and position screw were controlled.

### 3.1. Experimental Setup

**Figure 6.**
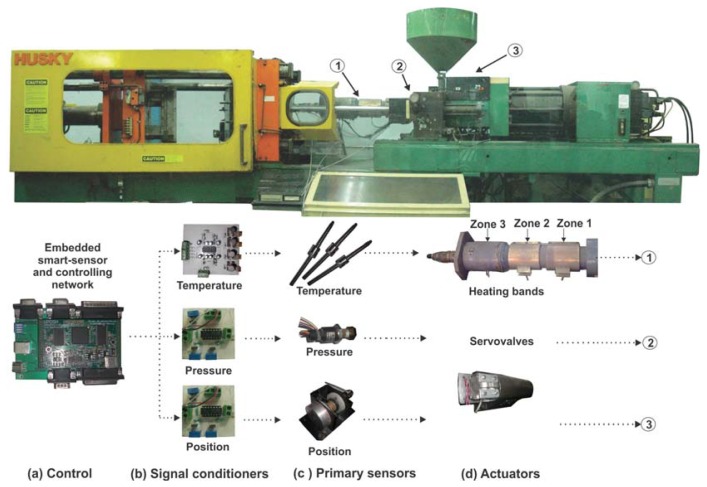
Experimental setup.

The experiments were carried out in a retrofitted HUSKY XL520RS injection molding machine as depicted in Figure 6; where the PIM machine, the embedded smart-sensor and controlling network, signal conditioners, primary sensors and actuators, can be seen. The FPGA unit was a proprietary Spartan 3E XC3S1600 platform (Figure 6(a)) and 30% of its resources were occupied by the proposed architecture after compilation. According to the variable to be controlled, three uIS processors were configured. In the first processor the following devices and parameters were used: an AD596 signal conditioner (Figure 6(b)); a type J thermocouple (Figure 6(c)), a sampling period of 200 ms, an oversampling-decimation factor of 200, and linearizing uses type J polynomial coefficients in Equation (1). Additionally, a low-pass FIR rectangular window filter of 16th order with an output saturation limit of ±2,047, and a PWM output were used in order to control three heating zones (Figure 6(d)).

The second processor was configured to control the injection pressure using the following attachments: a MLH03KPSB06A sensor pressure from Honeywell, with ±0.25% full scale accuracy and 200–3,000 psi pressure range, (Figure 6(c)) located in the PIM machine, an ADS7841 signal conditioner 12 bits resolution; a sampling period of 1 ms and the PWM used has a resolution of 12 bits.

### 3.2. Temperature Control

The temperature in the PIM process is one of the most studied variables [4,6,7,15,28] and its main problem is the coupling among the heating zones [4]. In this work a micro fuzzy logic controller (uF) is used to reduce the coupling effects among different temperature zones.

For the three different uFs utilized in this work, the input membership functions use five sets: negative large (NL), negative small (NS), zero (Z), positive small (PS), and positive large (PL). The output membership can be, depending on the variable to control, the following sets: negative very large (NVL), negative large (NL), negative medium (NM), negative small (NS), negative (N), zero (Z), positive (P), positive small (PS), positive medium (PM), positive large (PL) and positive very large (PVL). For the temperature controller, Table 2 shows the membership functions and the inference set of rules for the system based on the expertise of a human operator.

**Figure 7.**
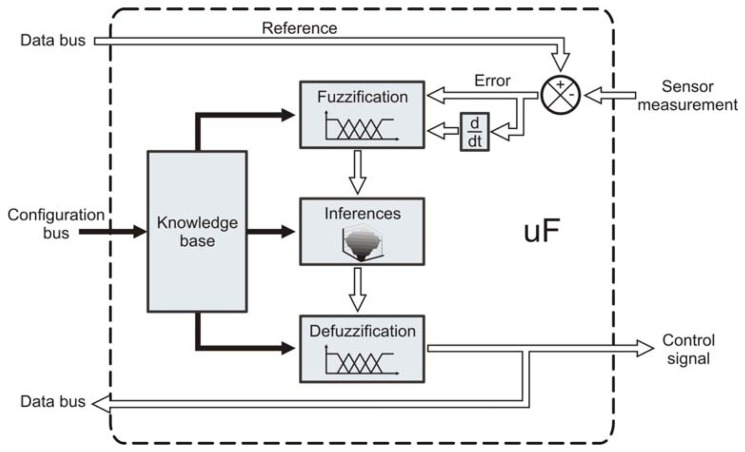
Temperature control test for different zones, the tests shows each zone with a different set point to evidence the decoupling.

The test for the temperature system consists in setting different temperature set points for each zone. The coupling effect is minimized by the control, as Figure 7 shows, where at the same time zones 1 to 3, were controlled. Even though the four zones use the same actuator, the dynamic behavior is different for each zone mainly due to its thermal load and the coupling effect. Since zone 3 has the largest load, it is the slowest; zone 2 “feels” the effect of zone 1 and zone 3, being the fastest. Finally zone 1 has the smallest load having an initial overshot of 10 °C. Once the transient stage was finished, the steady state error is below ±2 °C for all zones that overcome the coupling defect.

### 3.3. Injection Pressure Control

It is very important to control the injection pressure. If the injection pressure is too high, it is possible that the mould cavity will expand, causing material overflow. On the other hand, if pressure is too low, geometric defects and mechanical properties will appear in the finished product [3]. On a PIM machine the injection pressure is given by the injection screw. A hydraulic motor moves the injection screw operated by a servovalve. The injection pressure is hold while plastification or cooling begins, based on the type of materials (thermoset or thermoplastic) used. Due to the non-linearity of the injection pressure during compression a micro fuzzy logic controller (uF) is applied.

Table 3 shows the fuzzy set rules implemented for the pressure controller. The same membership functions for the error and error derivative used for fuzzy temperature controller are presented. The output membership functions, except its distribution, are similar too.

**Figure 8.**
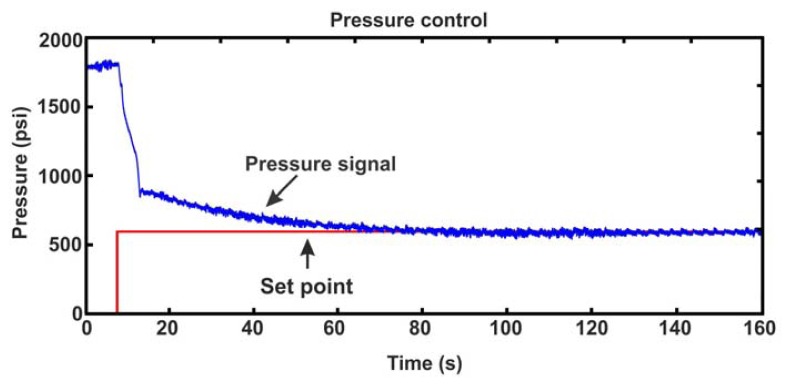
Pressure control test showing the reference and sensor measurement.

The fuzzy control behavior is shown in Figure 8. First, the injection pressure has an initial value of 1,800 psi, then; a reference of 600 psi is set. Interference appears when loading, because of the hydraulic accumulator which causes a delay of 5 s approximately. Finally, the controller reaches the reference in 70 s with an error in steady state below 3%.

### 3.4. Position Control

The screw position in a PIM machine is important to control the quantity of material injected into the mold. The selected PIM uses a hydraulic motor to move the screw that is operated by a proportional electrovalve. In this particular case the system response presents significant static friction and a delay because the valve activation is delayed 0.5 s; in addition, the hydraulic accumulator produces interference when loading. Therefore, classical PID controller becomes inefficient with this delay and with the friction non-linearity but a fuzzy controller is more robust managing this kind of systems.

Table 3 presents the inference rules for five membership functions of the error and its derivative. The input membership functions use the same five sets as the other controllers. Also, the output membership sets are shown.

**Figure 9.**
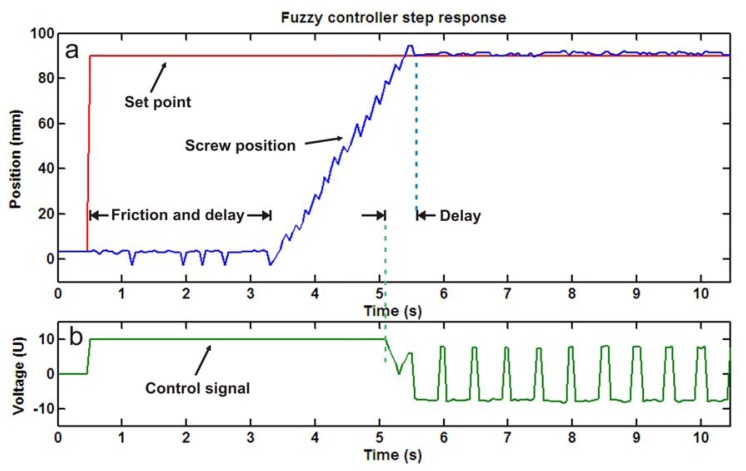
Screw position control test showing (**a**) the reference and the sensor measurement; (**b**) the control signal.

The embedded micro fuzzy logic controller programmed by the membership functions and inference rules of Table 4, behaves as Figure 9 shows. Figure 9(a) depicts the step response to a reference of 90 mm in the screw displacement with its characteristic delay of 0.5 s having an overshoot of 4% and a steady error below 2%. Figure 9(b) shows the reduction of the control signal before the position reaches the reference to limit the system overshoot due to the system delay. Finally, the position reaches the reference in 5 s without steady state error, a minimal overshoot overcoming the friction non-linearity and delay time in the system.

### 3.5. Validation of the Smart-Sensor Network

**Figure 10.**
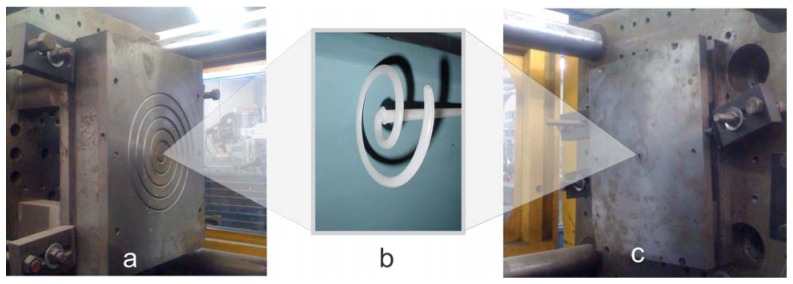
Example of an injection process with smart sensor (**a**) Archimedes spiral mold. (**b**) injected sample. (**c**) mold sprue.

In order to validate the proposed architecture, the aforementioned parameters were taken into account to control temperature, injection pressure and screw position in a particular experiment as shown in Figure 10. Using a mold based on Archimedes spiral [29] (Figure 10(a)), an injected sample is obtained as depicted in Figure 10(b). Considering its physical properties, the high-density polyethylene (grade PEAD 60120) was chosen and melted in heater sections; then, it was injected in the spiral mold through the mold sprue (Figure 10(c)). The injected sample obtained shows good appearance without deformations or apparent damages.

## 4. Conclusions

This work presents an FPGA-based smart-sensor network applied to injection molding machines. The main contribution of this work is to retrofit control in an injection molding machine, where the proposed architecture includes a smart-sensor processor, a programmable logic controller, several micro fuzzy logic controllers and a microprocessor network in a single chip. Due to the development of this system in a low cost device with high speed performance and wide capability to synchronize multiple processes, it was possible to obtain a collaborative sensor microprocessor with high-performance computational capabilities. Even though the system is capable of managing any variable in the PIM process, it was applied to control three of the most important variables that influence the product quality in injection molding machine: temperature, pressure and position. The temperature control tests in three different zones, the pressure control and position control, show good results, being the steady state errors below 3%. The application of the proposed system can be suitable to control retrofitted industrial machinery based on FPGA devices.

## Figures and Tables

**Table 1.** Resources usage.

**Unit**	**LUTs**	**BRAM**	**SLICES**	**MULT18×18**
uF	908	0	615	4
uIS	1,115	1	870	2
PLC	2,636	2	1,410	0
ioP	24	0	15	0
System manager	213	0	117	0
CP	1,105	0	788	0
Total	6,001	3	3,815	6
Available	14,752	36	29,504	36

**Table 2.** Fuzzy set rules implemented for the temperature controller.

**Output membership**		**Error derivative**				

		**NL**	**NS**	**Z**	**PS**	**PL**
**Error**	**NL**	NM	NM	NM	PM	PM
	**NS**	NM	NM	NM	Z	P
	**Z**	NM	Z	Z	P	P
	**PS**	NM	NM	N	PM	PM
	**PL**	NM	NM	Z	PM	PM

**Table 3.** Fuzzy set rules implemented for the pressure controller.

**Output membership**		**Error derivative**				

		**NL**	**NS**	**Z**	**PS**	**PL**
Error	NL	NM	NM	NM	N	Z
	NS	NM	NM	N	Z	P
	Z	NM	N	Z	P	PM
	PS	N	Z	P	PM	PM
	PL	Z	P	PM	PM	PM

**Table 4.** Fuzzy set rules implemented for the position controller.

**Output membership**		**Error derivative**				

		**NL**	**NS**	**Z**	**PS**	**PL**
Error	NL	NVL	NL	NM	NS	Z
	NS	NL	NM	NS	Z	PS
	Z	NM	NS	Z	PS	PL
	PS	NS	Z	PS	PM	PL
	PL	Z	PS	PM	PL	PVL

## Appendix

**Notation**	
*a_n_*	polynomial coefficients
G(s)	first order filter
H(z)	discrete transfer function
*k_f_*	filter gain
*T*	sampling period
*w_c_*	cut-off frequency
*x(k)*	input signal
*y*	nested polynomial
*y(k)*	filter output
